# Edge Computing and Blockchain in Enterprise Performance and Venture Capital Management

**DOI:** 10.1155/2022/2914936

**Published:** 2022-07-20

**Authors:** Zeyu Wang, Jia Lu, Mingyu Li, Siting Yang, Yutong Wang, Xin Cheng

**Affiliations:** ^1^School of Public Administration, Guangzhou University, Guangzhou, Guangdong 510006, China; ^2^Imperial College Business School, Imperial College London, South Kensington Campus, London SW7 2AZ, UK; ^3^Institute of Quality Development Strategy, Macro-quality Management Collaborative Innovation Center in Hubei Province, Wuhan University, Wuhan City 430072, China; ^4^School of Foreign Languages, Changchun University of Science and Technology, 7989 Weixing Road, Changchun City 130000, China; ^5^School of Politics and Public Administration, Wuhan University, Wuhan City 430072, China; ^6^School of Public Administration, Zhongnan University of Economic and Law, 182 Nanhu Road, Wuhan City 430070, China

## Abstract

Aiming at improving the enterprise performance and venture capital management, the edge computing is combined with blockchain technology in the construction of the supply chains so as to improve the stability and security of raw material supply chains of venture capital enterprises. Firstly, the relationships among venture capital, enterprise performance, and supply chain integration are analyzed, and the positive impacts of optimization and innovation of supply chain technology and venture capital on enterprise performance improvement are discussed. Secondly, the edge computing and blockchain technology are adopted to optimize the supply chain, and a protocol model is established to improve the security of supply chain information transmission. In addition, the supply chain Internet of Things (IoT) system is designed in layers based on blockchain and edge computing technology. Finally, the designed protocol model is applied to the supply chain and the supplier performance is evaluated by using the fuzzy analytic hierarchy process (FAHP). The results show that the gas value of the designed protocol model is stable at 104,000∼116,000 in blockchain transactions, and the execution time of a single blockchain transaction fluctuates between 4,300 and 6,100 ms. Therefore, the designed protocol model can effectively ensure the security of supply chain data during the transmission. The evaluation results of supplier performance suggest that the cooperative relationship between venture capital enterprises and suppliers is affected by many factors. Optimizing the supply chain structure and production activities can improve the performance of suppliers and venture capital enterprises. This work provides a reference for analyzing the performance of blockchain technology on venture capital enterprises and suppliers.

## 1. Introduction

With the development of capital market, venture capital has gradually become an important way of corporate financing. Venture capital is also a kind of equity capital invested by professional financiers in emerging industries, and its targets are mostly small and medium-sized high-tech enterprises, so it will increase the complexity and uncertainty of enterprise funds and management [1, 2]. Under the background of globalization and economic integration, the improvement of enterprise performance not only depends on the internal management mode of the enterprise but also needs to pay attention to the concentration of the supply chain, effectively integrate the external resources of the enterprise and realize the healthy competition of transactions among enterprises. The development of globalization has brought opportunities for the extension of high-tech enterprises and brought more intense competition and challenges to the growth of enterprises. The traditional production process of enterprises needs to go through the process of procurement, production, and market [3]. However, with the development and improvement of Internet technology and transportation and logistics, the scope of procurement of raw materials for enterprises has expanded. Enterprises need to find production raw materials and labor costs suitable for their own development from different regions and markets around the world. The cooperation among high-tech enterprises is due to the different needs of different enterprises, which are caused by their own enterprise development strategies and the lack of their own resources. Therefore, to reduce the time cost and opportunity cost of resource integration, the concept of supply chain integrated management is proposed to realize the centralized management of the enterprise itself, upstream, and downstream industries; and by coordinating cross-enterprise operation management, it can make full use of the benefits of enterprises in the context of globalization and eliminate risks in the supply chain [4].

Bessière et al. analyzed the development status of different types of venture capital raising platforms such as angel investment and directional investment and discussed related cases by discussing the financing trajectory and risk management dynamics. In addition, it analyzed the specific financing trajectory of a technology company that combines various crowdfunding methods and quantitatively explored the impact of initial crowdfunding activities on subsequent fundraising. It was found that the initial financing risk characteristics, financing trajectory, and follow-up activities have important impacts [5]. Kim explored the leadership style and management strategy of small and medium-sized venture companies. The empirical results showed that the leadership style of the management team has an important impact on the management strategy and will have a positive impact on the management strategy from the strategic direction, strategic control, organizational culture, and other aspects. Management strategies implemented by management are a factor in improving organizational performance, so the ability to implement management strategies should be enhanced. Therefore, the organizational performance of small and medium enterprises is a key factor for management to implement strategic leadership and management strategies [6]. Vedula and Fitza investigated the impact of venture capital and regional entrepreneurial ecology on startup performance by comparing the allocations associated with high-level performance of early-stage startups and mature late-stage startups. It was found that startup maturity is inversely related to the combined complexity of the regional factors that make up a regional entrepreneurial ecosystem and startups backed by venture capital gain the most from regional entrepreneurial ecosystem resources. In addition, it demonstrated the dynamic process of regional resource dependencies over the life cycle of startups [7]. Kim et al. analyzed the impact of technological connections formed between new and existing firms on firm development and found that when new firms have social connections with opportunistic incumbents, the technological connections between them can seriously hinder the formation of corporate venture capital deals, while when new firms have social ties with established firms that lack opportunity-prone, technological ties will facilitate the formation of corporate venture capital deals, because social ties strengthen firms' trust and provide more effective cooperation potential [8]. The current research suggests that the combination of edge computing and blockchain technology and the use of IoT technology to increase its application range can promote the construction capabilities of blockchain and edge computing as a shared database and data computing platform, respectively, provide users with end-to-end information services, and meet the application needs of modern intelligent development. To analyze the impacts of applying the blockchain technology in supply chain on enterprise performance and venture capital management, edge computing is adopted to optimize blockchain technology and is applied to the supply chain of venture capital enterprise suppliers. Then, an IoT system architecture based on edge computing combined with blockchain technology is established. By analyzing the impact of venture capital and supply chain on enterprise performance, the changes in supplier performance under the application of blockchain technology are analyzed. In addition, a supplier performance evaluation system is established under the venture capital enterprise and is assessed with the fuzzy analytic hierarchy process (FAHP) to verify the impacts of edge computing combined with blockchain technology on the performance of suppliers and venture capital companies.

## 2. Impacts of Edge Computing Combined with Blockchain Technology on Venture Capital Corporate Performance

### 2.1. Integration of Venture Capital, Corporate Performance, and Supply Chain

With the development of global economic integration, market competition has become increasingly fierce. Venture capital, as an important financing method for emerging companies, has become the main force to promote the improvement of corporate performance. The venture capital management can be taken as an important way to maintain the healthy development of enterprises, so as to accelerate the improvement of business performance, which is a key point to be considered for current researches [9–11]. Venture capital require to evaluate the capabilities and future development of the company and intervene in the management, operation, and supervision process of the invested company to provide effective help for emerging companies based on its own social resources and development experience. The high and economic profits can be obtained by means of equity transfer, and then the funds can be invested into new corporate projects to achieve a healthy operation of capital operations. Corporate performance refers to the operating efficiency and operator performance obtained by an enterprise during a certain operating period. Operating efficiency refers to the corporate development ability obtained through evaluation indicators such as operating indicators, debt solvency, and growth; operator performance refers to the contribution of the operator to the business and development of the company [12–15].

The corporate performance is mainly measured using the financial performance method and the market performance method. The financial performance method is to measure the impacts of the development strategy, operating capability, debt solvency, and profitability on the business performance goals of the company based on the return on assets, the return on net assets, and the growth capacity of the main business and other corporate operating profit indicators [16]. The market performance method uses Tobin's *q* value to measure the market value/asset replacement cost of an enterprise, but it is difficult to obtain market performance indicators. Therefore, the financial indicators are commonly adopted to evaluate and analyze quantitatively the corporate performance [17]. Relevant research shows that venture capital and corporate performance are positively correlated, which can promote the sound operation of business management, ease the corporate capital flow pressure, and broaden corporate financing channels, improving the corporate performance.

Venture capital requires a large amount of professional knowledge, rich management experience, and market development experience to provide effective suggestions for enterprise innovation, enhance the technological innovation capabilities of the company, and drive the further development of corporate performance [18]. From the perspective of fundraising, venture capital also provides financial support for the technological research and development of high-tech enterprises by enhancing corporate investment in scientific and technological research and development, optimizing the structure of talents, and introducing new production techniques [19]. From the perspective of the development interests of the company, venture capital can strengthen the corporate technological innovation and supervision, improving the corporate performance [20].

Supply chain management is to quickly adjust the upstream and downstream of the company through its own organizational method and timely reflect and adjust the market supply and demand relationship, so that the company can positively response and adjust the necessary procurement, production, and sales based on the needs of customers. The integration of supply chain requires unified management of supply chain enterprises and their affiliated resources, breaking through the boundaries of enterprises under certain economic effects, and realizing cross-enterprise cooperation and competition. For example, more mature high-tech companies such as Apple, Huawei, and Xiaomi focus on supply chain management in the external integration of the supply chain and form a fixed cooperative relationship with the upstream of the supply chain of the enterprise to further control the production cost and production period of the enterprise, so as to achieve the profit maximization of the company.

### 2.2. Impacts of Edge Computing and Blockchain on the Performance of Supplier

The blockchain technology is the core technology extracted from the digital currency led by Bitcoin. Its advantage is reflected in decentralization, and it achieves decentralized point-to-point transaction process in a distributed system through formula cryptography, timestamps, formula algorithms, and incentives, improving the efficiency of data processing and reducing the cost of data processing. As a shared distributed database, supply chain technology can effectively record the transaction information and data of both parties to the transaction, enhance the transparency and traceability of information, and ensure data security and information transmission efficiency [21]. The use of blockchain technology in the supply chain can greatly enhance the supply chain activities, quickly integrate the supply chain process, improve the data sharing requirements and inventory management capabilities of supply chain, standardize the transmission process of all data files, and elevate the reliability of supply chain decision-making. On the other hand, the applicability of blockchain technology on supply chain also shows that the technical advantages of blockchain technology have improved the performance level of supply chain [22].

From the perspective of supply chain resource management, supply chain can identify and analyze the unique resources and technical advantages of blockchain to help suppliers maintain a competitive advantage. Information sharing and supply chain trust are important ways that blockchain technology has a positive impact on supply chain performance. Information sharing can reduce the problems caused by information asymmetry and make supply chain adapt to market changes more quickly. The establishment of supply chain trust can promote the investment in technology and resources of both parties and improve the process efficiency and performance level of supply chain. Therefore, supply chain trust is a prerequisite for enterprise risk-taking [23]. In this study, it is proposed to use edge computing to optimize the blockchain technology and establish a supply chain protocol model to improve the use of blockchain technology in supply chain.

Edge computing is an open platform established by network technology, computing, and storage technology at the end close to the data source to provide data users with the closest end service. Since the response terminal of edge computing is close to the service initiator, it can provide faster network response services and meet the requirements of supply chain for real-time business processing and security and privacy. Compared with traditional cloud computing, edge computing shows the following advantages: (1) Low latency and high real-time performance. The edge device is close to the data source, which can process data in time, reduce unnecessary data transmission delay, and timely feedback to the next process after processing the data [24]. (2) Reduction of network bandwidth and power consumption. When the data is processed by edge devices, it can be distributed to nearby devices, which greatly reduces the consumption of network bandwidth and the computing load and power consumption of edge devices. (3) High security and fault tolerance rate. The edge computing process reduces the risk of data storage centralization and avoids data security problems in the channel [25].

**Figure 1:**
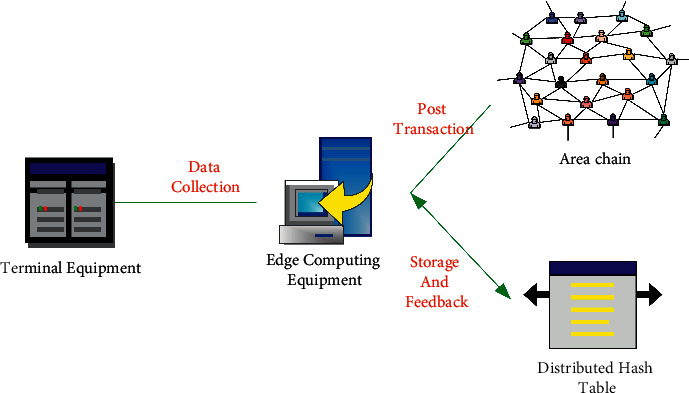
The protocol model based on blockchain technology and edge computing.

Based on edge computing and blockchain technology, the supply chain protocol is designed by combining with the cryptographic system to improve the security of supply chain information data transmission, thereby improving the information processing efficiency and privacy security of terminal equipment in the supply chain Internet of Things (IoT). The designed protocol model is shown in Figure 1.

The designed protocol model includes three parts: Terminal equipment, edge computing equipment, and blockchain. Terminal equipment can generate or integrate supply chain information, which are mainly sensors and intelligent terminal equipment with data collection functions. The equipment shows poor computing power and cannot perform complex computing processes, so the terminal equipment is required to transfer data to the edge computing device according to the protocol. The edge computing device is featured with low data transmission delay and strong computing power. It can quickly calculate the supply chain, help the terminal device to generate encrypted data, and back up the corresponding data [26–28]. Each piece of data will be stored in the blockchain to achieve the functions of decentralization, tamper-proof, and traceability, thereby ensuring the security of supply chain data. During the protocol, a pseudoidentity can be generated for the terminal device to realize anonymous storage, and the true identity can be reversed from the pseudoidentity when necessary. Therefore, the mapping relationship between the pseudoidentity and the real identity has to be protected through the encryption key generation link.

In addition, the supply chain information generated by the terminal device is also encrypted and stored, so the designed protocol can effectively and continuously protect the supply chain information data generated by the supply chain terminal device, and the security of data storage is use enhanced by encrypting the data. For the protocol model based on edge computing and blockchain, each node in the blockchain network has to process the data during the blockchain decentralization, which reduces the maintenance cost of the central server. Therefore, users are required to pay a certain amount of gas to the nodes that process data on the blockchain.

### 2.3. Evaluation on Performance of Supplier for the Venture Capital Enterprise

The evaluation of supplier performance is to regularly monitor and evaluate the performance of the upstream suppliers of the company's production, accurately evaluate the supply performance and cooperation between suppliers and enterprises, and further improve the production cooperation between venture capital enterprises and suppliers. During the evaluation of supplier performance, it should ensure timely communication and feedback. When the supplier performance is evaluated, it should consider the investigation and summary of interviews with relevant industry experts. In this work, based on the principle of objectivity and key points, the supplier performance is comprehensively evaluated, and a three-level evaluation index system is established at the target level, the criterion level, and the program level [29]. The evaluation indexes are refined layer by layer.

The evaluation indicators are selected based on the summary of relevant literature and experts' suggestions, and the FAPH is used to fuzzy process the evaluation indicators to establish a comprehensive evaluation model of supplier performance. The established supplier performance evaluation index system is shown in Table 1.

In the evaluation of the cooperative relationship between suppliers and venture capital enterprises, there are some qualitative indicators and some quantitative evaluation indicators. Therefore, traditional evaluation methods cannot meet the needs of supplier evaluation. FAH can be used to qualitatively describe and quantitatively analyze the fuzzy in the evaluation, and establish a comprehensive evaluation method for use and supplier performance evaluation [30].

During the fuzzy evaluation, a comprehensive evaluation indicator system has to be determined. In the evaluation on performance of supplier, *U* represents the evaluation indicator system, which could be written as follows:

(1) $$\begin{array}{c} U=\left(u_{1},u_{2},...,u_{i},...,u_{n}\right). \end{array}$$

*u* represents a specific evaluation indicator.

The comment set is a collection of comprehensive evaluation results made by experts in related fields on the evaluation object. It is represented by *V*, and its equation is expressed as follows:

(2) $$\begin{array}{c} V=\left(v_{1},v_{2},...,v_{i},...,v_{n}\right). \end{array}$$

*v* refers to the evaluation level of the evaluated object.

Since the importance of each evaluation indicator in the evaluation system for performance of supplier is different, it is necessary to assign the weight of the evaluation indicator according to the actual situation, and each evaluation indicator *U* is assigned corresponding weight *W*. *W* represents the set of evaluation indicator weights, which can be expressed in the following equation:

(3) $$\begin{array}{c} W=\left(w_{1},w_{2},...,w_{i},...,w_{n}\right). \end{array}$$

*w* represents the corresponding weight of the evaluation indicator.

A comprehensive evaluation matrix of the evaluation indicator system is established with

(4) $$\begin{array}{c} R=\left(r_{ij}\right)=\left[\begin{array}{cccc} r_{11} & r_{12} & \cdots & r_{1n}\\ r_{21} & r_{22} & \cdots & r_{2n}\\ \vdots & \vdots & \vdots & \vdots \\ r_{m1} & r_{m2} & \cdots & r_{mn} \end{array}\right]. \end{array}$$

*r* represents the judgment matrix in which the evaluation indicator *u* is rated as *v* level.

Fuzzy comprehensive evaluation is to solve the comprehensive evaluation results through fuzzy changes when the evaluation matrix and weight are known, and then normalize the evaluation scores. The calculation equation is given as follows:

(5) $$\begin{array}{c} B=W\cdot R\cdot V^{T}. \end{array}$$

*B* represents the fuzzy comprehensive evaluation score, *R* refers to the evaluation matrix, and *V*^*T*^ represents the fuzzy evaluation vector.

In this study, a venture capital company located in the Industrial Park of Changping District, Beijing is selected as the research object. The company has a professional technical team, a research & development (R&D) team, two generation factories, and two standardized production workshops. The company has established the strategic partnerships with many well-known multinational companies in the world. In the study, supplier A is selected as the research object for the evaluation on performance of supplier, and the established evaluation system for performance of supplier is applied for fuzzy comprehensive evaluation.

## 3. Impacts of Protocol Model on the Performance of Supplier

### 3.1. Performance Test of Protocol Model

**Figure 2:**
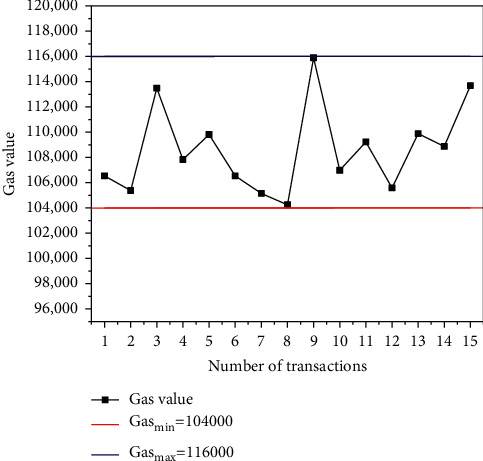
The gas value for transaction based on edge computing combined with blockchain technology.

In this work, web services are used to develop protocol models, and Web3j dependency packages are used to realize the interaction between web services and blockchain. The amount of information processing in the supply chain is tested and the gas value of information processing is recorded. Gas is a unit used to measure the computational effort required to perform a specific operation on the Ethereum blockchain. The result is shown in Figure 2.

Figure 2 illustrates that when different information is processed with the protocol model based on edge computing combined with blockchain, the required gas value is different. The reason is that the consumption of gas depends on the storage of data in the blockchain, so the consumed gas value of different data blocks is different. But overall, the gas value in blockchain transactions fluctuates between 104,000 and 116,000, so it can be considered that the amount of gas required for blockchain withdrawals for data processing is relatively stable.

**Figure 3:**
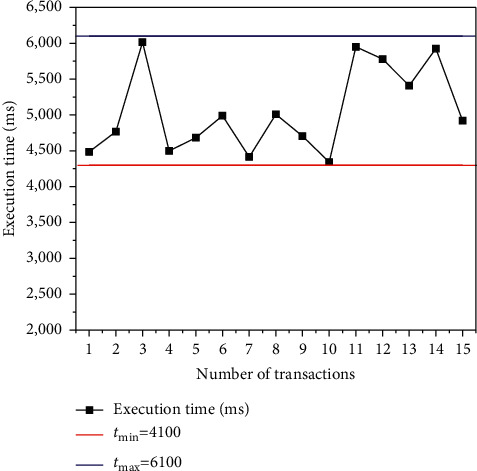
Execution time of the protocol model based on edge computing combined with blockchain technology.

The transaction execution time is tested to verify the data processing execution time of the protocol model based on edge computing combined with blockchain technology. The test results are shown in Figure 3.

Figure 3 reveals that the execution time of a single blockchain transaction fluctuates within 4300 ms to 6100 ms, and the overall consumption time of the protocol model shows a downward trend after multiple blockchain transactions, but the change trend is relatively not obvious. The reason is that the computer performance and quantity in the blockchain transaction process are limited, so the increase in the number of transactions will affect the increase in transaction time. Therefore, the designed protocol model based on edge computing and blockchain technology can ensure the security of data privacy during data transmission and shows the characteristics of tamper-proof and traceability, which further ensure the security of the supply chain system, improve the real-time performance of the system, and reduce the computing power consumption and data transmission bandwidth of IoT devices.

### 3.2. Determination of Evaluation Indicator System

According to the established evaluation indicator system for performance of supplier (Table 1), a comparison judgment matrix is constructed first, the weight is calculated, and the consistency is checked. 15 experts in the field are invited to quantitatively evaluate the importance of indicators for the evaluation indicator system using the 9-quantile ratio method. After construction of a judgment matrix, weight calculation, consistency check, and the weights for evaluation on performance of supplier are shown in Table 2.

Table 2 shows that the largest weight value of cooperative performance in the criterion layer is 0.41, followed by credibility and harmony, which are 0.21 and 0.18, respectively. Therefore, the product qualification rate and control of purchase cost are important factors that affect the performance of supplier. Therefore, suppliers need to pay attention to the product qualification rate of supply chain and strengthen the credibility of corporate cooperation in the process of communication with venture capital companies, so as to establish more continuous communicative and cooperative relationship.

### 3.3. Evaluation Results for Performance of Supplier

The performance of supplier A is evaluated by FAHP, and the evaluation index set is composed of 22 indicators that affect supplier performance. There are 5 evaluation results: excellent (5), good (4), medium (3), poor (2), and extremely poor (1), which form a supplier evaluation set. By combining the supplier's supply chain data such as the purchase records, delivery records, and delivery records of the venture capital enterprises; the performance relationship between the suppliers and the venture capital enterprises can be effectively reflected. In the fuzzy evaluation, the judgment matrix of qualitative indicators can be obtained through statistics. Its judgment matrix can be calculated by calculating the frequency of each evaluation level. The evaluation judgment matrix of a certain venture capital enterprise supplier in the Industrial Park of Changping District (Beijing) is shown in Table 3.

Facts have proved that the enterprise supply chain IoT system based on blockchain and edge computing can communicate with IoT devices in the supply chain, change the original supply chain management model, and establish a more efficient and reliable supply chain IoT application environment. This will improve the supplier's corporate performance and reduce the management risks that may exist in supply chain management. Equation (5) can be adopted to summarize the opinions of experts, and the comprehensive evaluation result of supplier A can be obtained as shown in the following equation:

(6) $$\begin{array}{c} A=\left[\text{0.}41\text{ 0.}21\text{ 0.}14\text{ 0.}18\text{ 0.}15\text{ 0.}13\text{ 0.}09\right]\left[\begin{array}{c} \begin{array}{c} \begin{array}{c} 2.13\\ 3.1355 \end{array}\\ 1.4055\\ 3.3880 \end{array}\\ 1.9655\\ 0.5510\\ 1.6660 \end{array}\right]=2.8584. \end{array}$$

The calculation results show that the evaluation results of supplier performance are between medium and poor. The fuzzy evaluation relationship matrix reveals that in the scheme layer, the scores of the evaluation indicators such as innovation ability, contract performance rate, risk and benefit sharing, management communication efficiency, information sharing, operation process reorganization ability, and professional knowledge imparting degree are scored lower which affects the performance of suppliers and negatively affect the cooperative relationship between venture capital enterprises and suppliers. Therefore, the suppliers of venture capital enterprises need to improve the performance of suppliers by optimizing the structure of the supply chain and production activities and establish a sustainable and healthy cooperative relationship.

## 4. Conclusion

According to the development characteristics of venture capital enterprises, a supply chain protocol model based on edge computing and block computing is established in this work, and it is applied to the supply chain operation of venture capital enterprises to improve the security and effectiveness of supply chain information transmission. In addition, the supply chain IoT system is designed based on edge computing and blockchain technology to meet the needs of enterprise supply chain management. To verify the validity of the application of the agreement model in the supply chain, the supplier performance under the venture capital enterprises is evaluated based on the FAHP, and the supplier performance evaluation index system is established from multiple perspectives. The experimental results show that the operation gas value and execution time of the designed protocol model are relatively stable which can effectively transmit supply chain information securely. The results of supplier performance evaluation show that the relationship between supplier A and the enterprise has a low score which affects the improvement of supplier performance and has a certain impact on the performance of the enterprise. Therefore, it is necessary for suppliers and venture capital enterprises to adjust the supply chain structure and establish a more healthy and sustainable cooperative relationship. However, there are still some shortcomings in this work. When the supplier performance is assessed, only 15 experts are found to evaluate, so there is a certain degree of subjectivity. In addition, the number of samples in this work is small, and the data set needs to be further optimized, which will be strengthened in the follow-up research.

## Figures and Tables

**Table 1:** Comprehensive evaluation system for performance of supplier.

Target layer	Criterion layer	Program layer
Evaluation on performance of supplier (A)	Cooperative performance (A1)	Product qualification rate (B1)
		Control of purchase cost (B2)
		On-time delivery rate (B3)
		Innovation ability (B4)
	Credibility (A2)	Willingness to cooperate (B5)
		Contract fulfillment rate (B6)
		Compliance (B7)
	Closeness (A3)	Proportion of purchase amount (B8)
		Contract period (B9)
		Risk and benefit sharing (B10)
	Harmony (A4)	Proportion of conflict events (B11)
		Communication efficiency of management (B12)
		Communication efficiency of staff (B13)
		Harmony of corporate culture (B14)
	Communication skills (A5)	Information sharing level (B15)
		Information response capability (B16)
		Status of production equipment (B17)
	Collaboration ability (A6)	Human resources investment level (B18)
		Capability of work flow reorganization (B19)
	Learning ability (A7)	Staff knowledge level (B20)
		Management level (B21)
		Professional knowledge transference (B22)

**Table 2:** Weights for evaluation on performance of supplier.

Target layer	Criterion layer	Weight layer	Program layer	Weight
Evaluation on performance of supplier (A)	Cooperative performance (A1)	0.41	Product qualification rate (B1)	0.34
			Control of purchase cost (B2)	0.25
			On-time delivery rate (B3)	0.16
			Innovation ability (B4)	0.09
	Credibility (A2)	0.21	Willingness to cooperate (B5)	0.53
			Contract fulfillment rate (B6)	0.29
			Compliance (B7)	0.45
	Closeness (A3)	0.14	Proportion of purchase amount (B8)	0.28
			Contract period (B9)	0.14
			Risk and benefit sharing (B10)	0.13
	Harmony (A4)	0.18	Proportion of conflict events (B11)	0.27
			Communication efficiency of management (B12)	0.09
			Communication efficiency of staff (B13)	0.67
			Harmony of corporate culture (B14)	0.33
	Communication skills (A5)	0.15	Information sharing level (B15)	0.41
			Information response capability (B16)	0.24
			Status of production equipment (B17)	0.14
	Collaboration ability (A6)	0.13	Human resources investment level (B18)	0.14
			Capability of work flow reorganization (B19)	0.08
	Learning ability (A7)	0.09	Staff knowledge level (B20)	0.16
			Management level (B21)	0.35
			Professional knowledge transference (B22)	0.13

**Table 3:** The judgment matrix for supplier evaluation.

Target layer	Criterion layer	Program layer	Excellent	Good	Medium	Poor	Extreme poor
Evaluation on performance of supplier (A)	Cooperative performance (A1)	B1	2	5	5	2	1
		B2	3	5	6	1	0
		B3	3	6	2	2	2
		B4	2	3	4	3	3
	Credibility (A2)	B5	4	2	6	2	1
		B6	2	4	6	0	3
		B7	1	4	8	2	0
	Closeness (A3)	B8	3	2	8	1	1
		B9	5	4	6	0	0
		B10	2	0	9	4	0
	Harmony (A4)	B11	1	4	7	2	1
		B12	2	5	4	3	1
		B13	2	3	8	2	0
		B14	2	3	10	0	0
	Communication skills (A5)	B15	3	5	3	3	1
		B16	2	4	7	2	0
		B17	2	0	10	1	2
	Collaboration ability (A6)	B18	5	2	6	0	2
		B19	4	0	6	2	3
	Learning ability (A7)	B20	5	1	9	0	0
		B21	5	1	7	2	0
		B22	0	4	6	3	2

## Data Availability

The data used to support the findings of this study are included within the article.
